# Role of *Candida albicans* in Oral Carcinogenesis

**DOI:** 10.3390/pathophysiology29040051

**Published:** 2022-12-07

**Authors:** Nurina Febriyanti Ayuningtyas, Fatma Yasmin Mahdani, Togu Andrie Simon Pasaribu, Muhammad Chalim, Visilmi Kaffah Putri Ayna, Arvind Babu Rajendra Santosh, Luigi Santacroce, Meircurius Dwi Condro Surboyo

**Affiliations:** 1 Department of Oral Medicine, Faculty of Dental Medicine, Universitas Airlangga, Surabaya 60132, Indonesia; 2 Oral Medicine Specialist Program, Faculty of Dental Medicine, Universitas Airlangga, Surabaya 60132, Indonesia; 3 Bachelor of Dental Science Program, Faculty of Dental Medicine, Universitas Airlangga, Surabaya 60132, Indonesia; 4 School of Dentistry, Faculty of Medical Sciences, The University of The West Indies, Mona Campus, Kingston JMAAW14, Jamaica; 5 Department of Medicine, School of Medicine, University of Bari, 70124 Bari, Italy

**Keywords:** *Candida albicans*, oral carcinogenesis, oral cancer, virulence

## Abstract

Oral carcinogenesis is also dependent on the balance of the oral microbiota. *Candida albicans* is a member oral microbiota that acts as an opportunistic pathogen along with changes in the epithelium that can predispose to premalignancy and/or malignancy. This systematic review uses the Preferred Reporting Items for Systematic Reviews and Meta-analyses guidelines to analyze the role of *Candida albicans* in the process of oral carcinogenesis. Eleven articles qualified inclusion criteria, matched keywords, and provided adequate information about the carcinogenesis parameters of *Candida albicans* in oral cancer. *Candida albicans* in oral carcinogenesis can be seen as significant virulent factors for patients with oral squamous cell carcinoma (OSCC) or potentially malignant disorder (OPMD) with normal adjacent mucosa. *Candida albicans* have a role in the process of oral carcinogenesis concerning morphological phenotype changes in cell structure and genotype and contribute to the formation of carcinogenic substances that can affect cell development towards malignancy.

## 1. Introduction

Oral squamous cell carcinoma (OSCC) is the most prevalent cancer in the oral cavity. Oral cancers with OSCC are found in more than 90% of cases [1,2]. OSCC is the fifth most common form of malignancy worldwide, along with oropharyngeal cancer [3]. More than 70% of deaths from this cancer occur in Asia, and two-thirds of cases occur in Asian countries such as Sri Lanka, Indonesia, India, Pakistan, and Bangladesh. Oral cancer is the most common malignancy, accounting for more than 25% of all new cancer cases yearly [4]. There is also a similarity to numerous factors aggravating the development of OSCC. Alcohol consumption and tobacco smoking are predisposing factors included in lifestyle factors alongside obesity and nutrient deficiency [5].

Moreover, other predisposing factors such as microbiome environment, genetic infirmity, environmental factors, and exposure to chemicals, pesticides, and heavy metals can induce pro-oncogenic genetic and epigenetic alterations, which lead to the development of OSCC [6,7,8]. In contrast to exogenous factors such as tobacco and alcohol, oral microbes in OSCC may result from the microbe being a commensal or secondary infection in the cancerous tissue [5]. It has been previously reported how the oral microbiota is involved in carcinogenesis by primarily paying attention to chronic inflammation, microbial synthesis of carcinogens, and alteration of the integrity of the epithelial barrier [9,10,11,12]. The human papillomavirus (HPV) has been widely discussed for its involvement in the development of oral cancer [13]. The presence of HPV and *Candida albicans* (as the normal flora) is able to contribute to the development of oral cancer [14]. Among the other species of *Candida, Candida albicans* has the highest prevalence found in OSCC and always gained concerns in relation to the ability of pathogenic state shifting from the commensal condition [15]. Hence, *Candida albicans* is considered to be one of the most commonly researched of oral microbes in OSCC [15]. Its commensal condition can develop into an opportunistic pathogen linked explicitly with the initiation of oral neoplasia and the development of OSCC [16,17,18,19]. It has also been reported that *Candida* invasion is a significant risk factor for the malignant transformation of oral potentially malignant disorder (OPMD) to oral cancer [20,21].

However, it has always been challenging to distinguish *Candida albicans* in the commensal or pathogenic state. In contrast, non-*Candida Albicans* species such as *Candida tropicalis* and *Candida glabrata* has been classified as pathogenic microorganisms, and their dominance in the oral cavity is considered a sign of oral dysbacteriosis. The shift in oral *Candida* species towards non-*Candida albicans* in OSCC may provide preliminary evidence for a potential pathogenic associate and OSCC [21]. Therefore, it is important to review several studies on the ability of *Candida albicans* to progress to a pathogenic state. Only a few studies still report that *Candida albicans* have a parameter by which the pathogenic form can be seen and compared to those where it is still commensal. The purpose is to reduce distortion from *Candida albicans* in pathological conditions aggravated by other predisposing factors. Therefore, from OPMD and cancerous lesions obtained in this review, it is essential to examine the type and prevalence of the lesion, which has been proven that *Candida albicans* is one of the factors in the development of oral cancer. It is also essential to identify the factors increasing the carcinogenesis capability of *Candida albicans.* Thus, role of *Candida* spp. in OPMD and malignant can be accurately correlated and these factors can be intervened. In addition, the antifungal therapy that can be administered will also be more specific to the treatment, increasing the curative effect for preventing synergistic role in carcinogenesis and patient survival rate up to 80% along with treatment of other predisposing factors when it is appropriately diagnosed at an early stage [4].

## 2. Materials and Methods

### 2.1. Data Sources and Search Strategy

The preferred Reporting Items for Systematic Reviews and Meta-analyses (PRISMA) were used in this systematic review. A comprehensive search of the scientific literature was carried out in the following databases: PubMed, Scopus, and Web of Science for the studies published from 1987 to 2022.

The titles and keywords used were a combination of the following keywords adapted to each database: [(“*Candida albicans*” OR “Oral Candidiasis”) AND (“Oral Carcinogenesis” OR “Oral Cancer” OR “OSCC” or “Oral Squamous Cell Carcinoma”) AND (“Oral Potentially Malignant Disorder” OR “OPMD”)]. The keyword searches were run from 30 December 2021, to 1 December 2022. The manual searching and Embase cross-referencing method are also used to complete the investigation.

### 2.2. Study Selection

All studies including case-control, analytical cross-sectional studies, cohort, and randomized controlled trials that evaluated the role of *Candida albicans* in oral carcinogenesis were included. The inclusion criteria were articles describing *Candida albicans* as a predisposing factor for oral carcinogenesis.

### 2.3. Data Extraction

Three authors screened each study independently (MC, VKPA, and TASP). Both MC and VKPA are last year of undergraduate dentistry program. The authors first screened the title(s), abstracts, and full texts to determine whether the inclusion criteria had been accomplished. The following information was extracted from the studies to be included in the systematic review: study design, oral cancer diagnosis, oral candidiasis diagnosis, and *Candida albicans* virulence marker. In case of disagreement, third investigators (NFA, MDCS, and FYM) acted as a referral and reach a consensus through discussion.

### 2.4. Data Synthesis and Analysis

The data extracted from included articles were classified into four group types of data: study characteristics, case characteristics, diagnosis procedure, and result. The reports were identified based on virulence markers of *Candida albicans* related to carcinogenesis (i.e., phenotype, genotype and metabolic identification). The samples must mainly be taken from patients with oral cancer or OPMD diagnosis.

## 3. Results

### 3.1. Study Characteristic

Most samples of oral carcinoma were defined as oral cancer, oral dysplasia, OSCC without lymph node metastases (LNM), OSCC regional LNM, and adenocarcinoma. The OPMD was defined as leukoplakia (homogenous and non-homogenous), erythroplakia, oral lichen planus (OLP) (reticular, plaque, atrophic, or mixed), atypical lichen planus, oral lichenoid lesion (OLL) (reticular, plaque, atrophic, or mixed), and oral submucous fibrosis (OSF) (Table 1).

### 3.2. Candida Isolation and Culture Procedure

Most samples of OSCC and oral cancer were performed through tissue biopsy, oral rinse, mucosal swab, and saliva collection to detect *Candida’s* presence. Most studies cultured the specimen by CHROM-agar medium as well as the Hematoxylin and Eosin (HE) staining, and examination under fluorescent microscopy for analysis of *Candida* morphology. The assimilation test, agglutination test, Leicester and real-time polymerase chain reaction (RT-PCR) to identify *Candida* species was also performed (Table 2).

### 3.3. Candida Virulence Factor

#### 3.3.1. Phenotype

The phenotype of *Candida* is defined as the presence of hyphae, spores, colonies, biofilm formation, and Cell Surface Hydrophobicity (CSH). Oral cancer and epithelial dysplasia showed a higher presence of *Candida* spp. than healthy gingiva tissue. Oral cancer itself showed higher colonies, biofilm formation, biofilm mass activity, and CSH (Table 3).

#### 3.3.2. Genotype

The genotype of *Candida* has defined as *Candida albicans* alcohol dehydrogenase 1 (CaADH1) mRNA gene was strongly expressed in OSCC and oral dysplasia and especially with LNM. The identified Candida was dominated by *Candida albicans* genotype A. The Stratifin (SNF) was highly expressed in the *Candida albicans* infection both OPMD and OSCC (Table 4).

#### 3.3.3. Metabolic Factors

The metabolic factors of *Candida* were defined as the production of acetaldehyde, proteinase, proteolytic, lipolytic, phospolytic, esterase, and N-nitroso benzyl methylamine (NMBA) production (Table 5).

## 4. Discussion

*Candida albicans* is the most virulent pathogen among other *Candida* species and is most commonly found in OSCC and OPMD patients [33,34]. However, *Candida albicans* is also an important component of the commensal microbiota in maintaining oral health and the general physiology of the human body and contributes to overall health [35]. Thus, even though this microbiota has been found in large numbers in healthy individuals with healthy oral cavities, the results found in comparisons between OSCC and healthy ones may be inconclusive and insignificant [5]. Therefore, to see the role of microorganisms in OSCC, research results with a highly significant difference between OSCC patients compared to healthy patients with normal oral mucosa are necessary [5]. Some of the articles reviewed in this systematic review are unable to show the course of the disease caused by patients with oral dysbiosis caused by a high prevalence of *Candida albicans* or by patients presenting the pathogenic phase of *Candida albicans* in the form of oral candidiasis and chronic hyperplastic candidiasis to OSCC [22,23,24,25,26,27,28,29,34]. Nevertheless, the role of *Candida albicans* in the process of oral carcinogenesis can still be seen from the importance of *Candida albicans* virulence factors generated between OSCC or OPMD patients compared to normal healthy mucosa [5,36,37,38].

In general, the role of *Candida albicans* in the process of carcinogenesis tends to be complex, such as the role of virulence factors, the host genome, influence on the immune response, and oral dysbiosis, as noted in a review conducted by Di Cosola et al. 2021 [35]. However, several studies have been conducted, and no direct study of *Candida albicans* with OSCC or OPMD [39,40,41,42,43,44,45]. In this systematic review, there are eight articles dominated by the *Candida albicans* virulence factor. In general, there are seven virulence factors of *Candida albicans* discussed in this article, mainly phenotype (*Candida* frequencies, hyphae, sphere, colonies, biofilm formation), genotype (*Candida albicans* alcohol dehydrogenase 1 (CaADH1) mRNA gene, genotypic diversity of *Candida albicans* strains, CSH), and metabolic production (acetaldehyde product, lipase, proteinase product, phospholipase, and esterase activity, NMBA production) [22,23,24,26,27,28,29,34].

Increased colonization of *Candida albicans* is one of the strong associations with oral epithelial dysplasia and neoplastic transformation leading to the OSCC process [46,47]. The number of colonies and excessive density of *Candida albicans* can damage host cells and promote the development of carcinogenesis [34]. The presence of *Candida albicans* in the form of colonies and biofilm formation found in the healthy mucosa group compared to moderate, severe dysplasia and OSCC showed high statistical significance. However, based on research conducted by Tamgadge et al., 2017 and Alnuaimi et al., 2016, the study did not concern *Candida albicans* in detail but rather *Candida* as a whole [22,29]. In addition, adhesiveness also influences the early stages of colony formation, one of which is CSH as a reference for measuring adhesiveness to hydrophobic substrates such as buccal keratinous tissue and oral prostheses [48,49]. Differences in CSH in all study groups (oral cancer, atypical lichen planus, chronic candidiasis) compared to healthy mucosa were statistically significant [26].

The genotypic diversity of *Candida albicans* in the oral cavity, with its relationship to high colonization in OSCC, means that variety may also influence the process of oral carcinogenesis [24,29,30,31,50]. At the subspecies level, only *Candida albicans* species have been extensively genotyped by the ABC genotyping method based on the size and presence of transposable introns in 25S ribosomal DNA in *Candida albicans* genotype A, genotype B, and genotype C [12,24,35]. *Candida albicans* genotype A strain observed in oral cancer patients had a significantly higher incidence compared to non-oral cancer patients. Furthermore, the genotype B *Candida albicans* strain found in non-cancer patients had a substantially higher incidence than in oral cancer patients [24]. However, regarding the ability to produce carcinogenic substances such as acetaldehyde, there is no significance between the individual genotypes of *Candida albicans* [29].

*Candida albicans* also have the potential to induce OSCC by producing carcinogenic compounds. Certain strains of *Candida albicans* and other yeasts play an essential role in developing oral cancer by creating endogenous nitrosamines. *Candida albicans* can convert both nitrite and/or nitrate into nitrosamines and other substances to produce acetaldehyde [25,50,51]. The *Candida albicans* strain showed the highest nitrosation potential compared to other strains. Strains with high nitrosation potential is generally isolated from lesions with more advanced oral precancerous changes [25]. However, it should also be noted that the study had a low-quality assessment of the risk of bias, including unclear details of the inclusion of research subjects and neglect of confounding factors that could affect study results [25]. *Candida albicans* has the ability to convert alcohol to acetaldehyde, which has carcinogenic role in oral cavity. This conversion is facilitated by *Candida albicans* Alcohol dehydrogenase 1 (CaADH1) mRNA gene [24]. The study results showed that the CaADH 1 gene could be significantly associated with OSCC with and without metastases compared to healthy patients but could not be predicted in patients with oral dysplasia compared to healthy patients. Studies examining the potential role of *Candida* and CaADH1 mRNA in the initiation and progression of oral dysplasia and OSCC and metastatic OSCC is relatively rare. In this study, not all isolates demonstrated the presence of the CaADH1 mRNA gene in *Candida albicans* [23].

One of the interesting points is the finding of SFN expression oral carcinogenesis [32]. SFN is a cell cycle checkpoint protein that has been reported to be involved in carcinogenesis [52]. In some cancers, such as ovarian cancer [53], breast cancer [54], ocular surface squamous neoplasia [55], and lung cancer [56], the SFN was significantly higher and determined as a prognosis [53]. The higher SFN expression is correlated with higher p53 expression [55]. In oral cancer, the SFN expression is influenced by *Candida albicans* [32]. Both OPMD and OSCC with *Candida albicans* expressed a higher SFN expression than those without *Candida albicans* and healthy mucosa [32]. The role of SFN in oral carcinogenesis is not yet fully understood. This discovery leads us to research more about the involvement of SNF in oral carcinogenesis.

*Candida albicans* acetaldehyde production occurs through the conversion of alcohol to carcinogenic metabolite acetaldehyde. Pure ethanol has no independent carcinogenic effects, but the first metabolite of acetaldehyde is an undeniable carcinogen. It can induce mutagenic impacts such as DNA adducts, cross-linking, aneuploidy, or chromosomal aberrations [24,57,58]. Acetaldehyde metabolism is significantly influenced by smoking behaviour and alcohol [59]. Therefore, these risk factors must also be included in the research variables of articles examining acetaldehyde production. All *Candida albicans* isolates were shown to produce high levels (>100 μM) of acetaldehyde in all incubations containing ethanol, ethanol–glucose or glucose incubation. The acetaldehyde-producing ability was significantly lower in OPMD lesions (OLP, OLL, and leukoplakia) than in non-cancer patients [27]. Among them, leukoplakia has acetaldehyde-producing ability higher than OLP and OLL [27]. This difference in acetaldehyde-producing ability is influenced by predisposing factors such as smoking and alcohol consumption. Where are the predisposing factors can induce adaptive changes that lead to the overregulation of acetaldehyde metabolism by *Candida albicans.* Hence, acetaldehyde production is influenced in candida mediated oral carcinogenesis [27].

*Candida albicans* can also affect the progression from OPMD to OSCC. Among other OPMD lesions, oral leukoplakia has the highest incidence and rate of malignant transformation. Oral leukoplakia and its association with *Candida albicans* may be associated with a higher risk of malignant transformation [22,27]. There is also a significant association between histologically oral mucosal fungal infections and epithelial dysplasia [25]. This is associated with increased exposure to other risk factors such as alcohol and smoking. Isolates from the oral leukoplakia group produced significantly higher levels of acetaldehyde than isolates from oral lichen planus (reticular, plaque, atrophic, or combination) when exposed to glucose–ethanol or glucose alone. In oral leukoplakia, higher *Candida* colonisation rates, higher smoking rates, and a cumulative increase in *Candida* acetaldehyde metabolism may contribute to malignant transformation [27]. Another study also confirmed that the leukoplakia with *Candida albicans* (or Candidal leukoplakia) has a high rate of cancer transformation, because it highly expressed a fibroblast activation protein (FAP) and α-smooth muscle actin (α-SMA). In addition, leukoplakia also secretes low a CX3CL1, a potential antifungal protein, which is more resistance to *Candida albicans* [60].

Factors affecting the ability of *Candida albicans* to accelerate the development of oral carcinogenesis also includes virulence factors, protein-degrading ability, and lipolytic activity. The persistence of *Candida* also depends on the capacity to secrete hydrolytic exoenzymes that facilitate further tissue invasion [26]. It has been shown statistically that *Candida albicans* proteinase, phospholipase and lipase activity was higher in oral cancer patients [26,28,29]. However, it was found that *Candida albicans* esterase activity was significantly higher in healthy patients than in oral cancer patients. In addition, in Castillo 2018, the research was not explicitly conducted on *Candida albicans* but on *Candida* in general [26].

**Figure 1:**
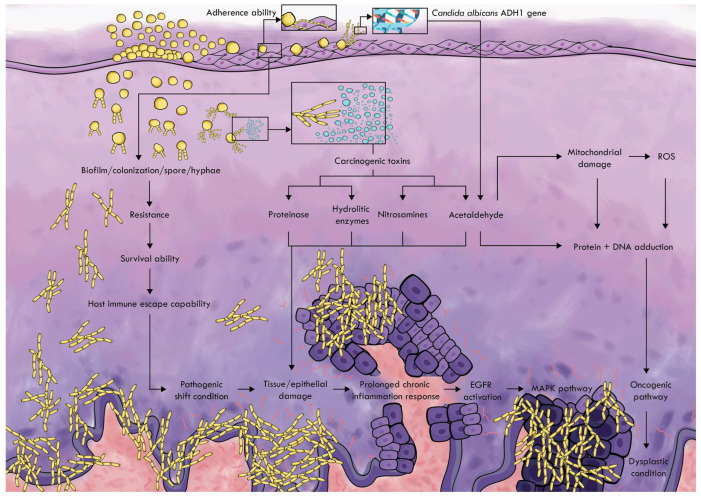
*Candida albicans* carcinogenic pathway.

*Candida albicans* can stimulate the origin and development of cancer. These studies examine several mechanisms by which this fungi type can trigger cancer. The more precise image of the mechanism is summarized as stated in Figure 1.

Adherence ability is the first step in colonization with *Candida albicans*. CSH is a method for determining the adherence ability of microorganisms to hydrophobic substrates, such as buccal keratin tissue and oral prostheses. This ability affects the formation of biofilms that contribute to maintaining the prolonged infection and avoiding the defense mechanism of the host [49,50]. *Candida albicans* in normal microbiota has a normal level of biofilm and colonization, while in the oral microbiota dominated by *Candida albicans*, the relationship between biofilm formation and colonization increases, which increases resistance and survival, which leads to invasive colonization and pathogenic shifting state [61,62]. The variety of *Candida albicans* strains also influences the ability of these microorganisms to form biofilms [29,63]. This leads to tissue damage and triggers a continued chronic inflammatory response [29,61,62].

Chronic contact with microorganisms and their products such as endotoxins, enzymes are toxic for host cells, which can either trigger mutations or alter the signaling pathways to influence on cell proliferation or the survival of the epithelial cells [64]. *Candida albicans* can produce carcinogens such as acetaldehyde, nitrosamines, and enzymes (proteases, lipases, esterases, and phospholipases) that can promote cancer formation [29,62,64,65].

One of the proteins identified in the mannoprotein infraction of *Candida albicans*, which increases tumor adhesion by triggering inflammation in endothelial cells, is alcohol hydrogenase (ADH1), which is associated with a cancer-stimulating mechanism by acetaldehyde production. *Candida albicans* use the enzyme alcohol hydrogenase (ADH1) to convert alcohol and other substances, such as carbohydrates into carcinogenic acetaldehyde [62]. This ability is encoded by the ADH gene, which is expressed in seven types of ADH genes in the genome database by *Candida albicans* [23]. Acetaldehyde can induce tumor development in various ways. This carcinogen binds to proteins and DNA, changes its structure and function, and the reduction in the antioxidant activity of glutathione increases the content of reactive oxygen species (ROS) in the cells. These changes can lead to genomic instability, inhibiting the apoptotic system and tumor development [62]. Nitrosamines produced by *Candida albicans* individually or in combination with other carcinogenic compounds can activate specific proto-oncogenes that can cause the development of cancer lesions that lead to changes in dysplastic conditions in oral epithelium and cancer [62,66]. Carcinogenic products and hydrolytic enzymes produced by *Candida albicans* also lead to further tissue destruction and trigger a continuous chronic inflammatory reaction [29].

Some of the results from these articles also show shortcomings that there are studies that are not being conducted specifically on the *Candida albicans* subspecies but focused on *Candida* species in general. However, *Candida albicans*, the most prevalent fungal microbiota in the oral cavity, are expected to be considered from the results obtained [33,34]. In addition, there are several articles that only list OPMD without OSCC; thus, a study group without OSCC can lead to different results [25,27]. However, from the significance obtained from the reviewed research, the comparison of OPMD with normal mucosa seems to be statistically significant, which will have a much higher importance in OSCC, whose histopathological and clinical condition is much more severe than in OPMD. Another thing to consider is the pathological state or condition of *Candida albicans*, which is difficult to determine when the microorganism is already pathogenic or still opportunistic commensal, characterized by the absence of articles containing oral candidiasis in the study group. However, some of the highly significant differences between OPMD or OSCC and normal mucosal patient groups must be considered when determining the role of *Candida albicans* in carcinogenesis.

## 5. Conclusions

*Candida albicans* were closely associated with oral potentially malignant and malignant oral lesions with various pathways. However, this systematic review study flags the important parameters involved in the oral carcinogenesis process, which include production of several phenotypes, genotype virulence factors, and carcinogenic metabolites.

In general, the relationship between fungal infections, especially *Candida albicans*, and oral cancer has been discussed in the literature for a long time. Many in vitro and in vivo studies show evidence of the parameters and markers involved in *Candida albicans* carcinogenesis. However, clinical evidence is still lacking, as it is difficult to find studies that deal specifically with this topic. Risk factors affecting the virulence factors of *Candida albicans* is also necessary to be researched in more articles involved. The exact mechanism by which *Candida albicans* is interested in developing OSCC also requires much research, particularly clinical research.

## Figures and Tables

**Table 1:** Sample characteristics in each study.

Country	Study Design	Case Groups (*n*)	Control Groups (*n*)	Reference
India	Retrospective analytic cross sectional	- Oral dysplasia (30) - Oral carcinoma (30)	Healthy gingiva (20)	[22]
Egypt	Retrospective analytic cross sectional	- Oral dysplasia (16) - OSCC without LNM (16) - OSCC with regional LNM (15)	Healthy gingiva (7)	[23]
Australia	Case-control	Oral cancer (52)	Healthy mucosa (104)	[24]
Denmark	Analytic cross-sectional	Leukoplakia and erythroplakia (12)	NA	[25]
Argentina	Analytic cross-sectional	- Malignant OSCC (25) - Atypical lichen planus (11)	Asymptomatic *Candida* spp. carriers with healthy mucosa (15)	[26]
Spain	Analytic cross-sectional	- OLL reticular type (2) - OLL reticular and plaque type (2) - OLL reticular and atrophic type (2) - OLP reticular type (2) - OLP reticular and plaque type (2) - OLP reticular and atrophic type (6) - OLR reticular, plaque, and atrophic type (10) - Leukoplakia homogenous (5) - Leukoplakia non-homogenous (1)	Healthy mucosa with positive *Candida albicans* (6)	[27]
Finland	Analytic cross-sectional	- OSCC (100) - Adenocarcinoma (3) - Other malignancy (3)	NA	[28]
Australia	Analytic cross-sectional	Oral cancer (52)	Non-oral cancer patients (104)	[29]
India	Analytic cross-sectional	- OSCC (97) - OPMD (200)	Healthy mucosa (200)	[30]
India	Prospective and observational study	- OPMD (leukoplakia, OLP, OSF) (50) - OSCC (50	Healthy mucosa (50)	[31]
Taiwan	Analytic cross-sectional	- OPMD ^ab^ - OSCC ^b^	Health gingiva ^b^	[32]

*n* = number; NA = not available; a = not describe the exact diagnosis; b = not mentioned the number of samples.

**Table 2:** The *Candida albicans* isolation and the diagnosis procedure.

Sampling Methods	Detection Methos	Reference
Tissue biopsy	HE staining then examined under fluorescent microscopy	[22]
Tissue biopsy	HE staining then examined under fluorescent microscopy	[23]
Oral rinse	Culture in CHROM-agar *Candida* medium	[24]
Tissue biopsy and swab	- Pure cultures of yeasts cultured on Candida BCG agar - *Candida albicans* strains were identified using the Leicester procedure	[25]
Swab	- Colony morphology - Biochemical tests	[26]
Swab	- Colony morphology - Growth of chromogenic agar - Microscopy - Biochemicals reactions in API ID32C assimilation tests	[27]
Saliva collecting	- Colony morphology on CHROM-agar *Candida* medium - Latex agglutination test - API ID 32C assimilation tests	[28]
Oral rinse	- CHROM-agar *Candida* medium - RT-PCR-high resolution to analyze the species	[29]
Saliva collecting	- Colony count	[30]
Swab	- Colony morphology on HiCrome Candida Differential HiVeg agar/CHROMagar	[31]
Tissue biopsy and swab	- Colony morphology on CHROM-agar *Candida* medium - Immunohistochemisty	[32]

NR = not reported.

**Table 3:** The *Candida albicans* phenotype identified in each lesion.

Phenotype Marker	Result	Reference
The presences of hyphae and spore	- The oral cancer and epithelial dysplasia showed a higher presence of *Candida* species hyphae than healthy gingiva tissue. - The oral cancer showed a higher number of *Candida* sp than epithelial dysplasia.	[22]
Colonies	- The oral cancer showed a higher number of *Candida albicans* colonies than healthy mucosa.	[24]
	- The OSCC showed higher a *Candida albicans* colonies than healthy mucosa. - The OPMD showed higher a *Candida albicans* colonies than healthy mucosa. - The OSCC showed higher a *Candida albicans* colonies than OPMD.	[30]
	- The OSCC showed higher a *Candida albicans* colonies than healthy mucosa. - The OSCC showed higher a *Candida albicans* colonies than OPMD.	[31]
Biofilm formation	- The oral cancer showed higher biofilm formation than asymptomatic carriers with healthy mucosa.	[26]
	- The oral cancer showed higher biofilm mass activity than non-oral cancer patient.	[29]
CSH	- The oral cancer showed higher CSH than asymptomatic carriers with healthy mucosa	[26]

**Table 4:** The *Candida albicans* genotype identified in each lesion.

Genotype Marker	Result	Reference
CaADH1 mRNA gene	- The OSCC and oral dysplasia showed higher expression than healthy normal gingiva - The OSCC with regional LNM showed higher expression than OSCC without LNM - The oral dysplasia showed lower ex gene than OSCC with regional LNM and OSCC without LNM	[23]
Strain genotypic	The oral cancer tissue is dominated by *Candida albicans* genotype A	[24]
SFN	- The OPMD with *Candida albicans* infection showed a higher SNF expresion than OPMD without *Candida albicans* infection and healthy normal gingiva - The OSCC with *Candida albicans* infection showed a higher SNF expresion than OSCC without *Candida albicans* infection and healthy normal gingiva - The OSCC with *Candida albicans* infection showed a higher SNF expresion than OPMD with *Candida albicans* infection	[32]

**Table 5:** The *Candida albicans* metabolic product identified in each lesion.

Genotype Marker	Result	Reference
Acetaldehyde	- The *Candida albicans* in OLL and OLP produce lower acetaldehyde than healthy mucosa ethanol incubations. - The *Candida albicans* in OLL, OLP and leukoplakia produce lower acetaldehyde than healthy mucosa in ethanol–glucose incubations. - The *Candida albicans* in OLL produce lower acetaldehyde than healthy mucosa in glucose incubations. - The *Candida albicans* in OLL and OLP produce lower acetaldehyde than leukoplakia in glucose incubations.	[27]
	- The oral cancer showed higher percentage of *Candida* with high acetaldehyde producing ability than non-oral cancer patients. - The oral cancer with *Candida albicans* showed higher acetaldehyde amount than non-oral cancer patients.	[29]
Proteinase	- The oral cancer with *Candida albicans* showed higher proteinase than non-oral cancer patients.	[29]
Proteolytic activity	- The OSCC showed higher proteolytic activity than asymptomatic carriers with healthy mucosa. - The atypical lichen planus showed higher proteolytic activity asymptomatic carriers with healthy mucosa. - The OSCC showed similar proteolytic activity with atypical lichen planus.	[26]
	The oral cancer with *Candida albicans* showed lower proteolytic activity than *Candida tropicalis* as non-albicans strain.	[28]
Lipolytic activity	- The oral cancer showed higher lipolytic activity than asymptomatic carriers with healthy mucosa. - The atypical lichen planus showed higher lipolytic activity than asymptomatic carriers with healthy mucosa. - Asymptomatic carriers with healthy mucosa showed completely absent of lipolytic activity.	[26]
Phospolytic activity	- The oral cancer showed higher phospholipase activity than non-oral cancer patients.	[29]
Esterase activity	- The oral cancer showed lower esterase activity than healthy patients.	[29]
NMBA production	The erythroplakia and leukoplakia with *Candida albicans* exhibited the highest nitrosation production.	[25]

NR = not reported.

## Data Availability

The data available is a personal request to the corresponding author (nurina-ayu@fkg.unair.ac.id).
